# Telemedicine in Eating Disorder Treatment: Systematic Review

**DOI:** 10.2196/74057

**Published:** 2025-11-17

**Authors:** Juan Antonio Blasco Amaro, Agnieszka Dobrzynska, Rebeca Isabel-Gómez, Enrique Enrique Perez-Ostos, Eva María Venegas Moreno

**Affiliations:** 1 AETSA, Andalusian HTA Office Fundación Progreso y Salud Seville Spain; 2 Preventive Medicine and Public Health Department Hospital Universitario Virgen del Rocío Sevilla Spain; 3 Endocrinology and Nutrition Management Unit Hospital Universitario Virgen del Rocío Sevilla Spain; 4 Instituto de Biomedicina de Sevilla Seville Spain

**Keywords:** telemedicine, telehealth, eating disorders, teletherapy, bulimia nervosa, anorexia nervosa, binge eating disorder, eating disorder not otherwise specified, EDNOS

## Abstract

**Background:**

Telemedicine has emerged as a promising tool to enhance adherence and monitoring in patients with eating disorders (EDs). Traditional face-to-face cognitive therapies remain the gold standard; however, integrating telemedicine may provide additional support and improve patient engagement and retention. Given the increasing use of digital health interventions, it is crucial to assess their safety and effectiveness in complementing conventional treatments.

**Objective:**

We aimed to evaluate the safety and effectiveness of telemedicine as a complementary tool for cognitive face-to-face therapies to promote adherence and monitoring of patients with EDs.

**Methods:**

We consulted the National Institute for Health and Care Excellence, the Canadian Agency for Drugs and Technologies in Health (now known as Canada’s Drug Agency), MEDLINE (Ovid), Embase, Web of Science, Cochrane Library, international HTA database (International Network of Agencies for Health Technology Assessment), CINAHL (EBSCO), and PsycINFO (EBSCO) websites and databases in December 2024 to identify eligible systematic reviews, synthesis reports, or meta-analyses that address telemedicine as a complementary therapy to face-to-face care in patients with EDs. Two researchers performed an independent critical reading of the systematic reviews and assessed the risk of bias using AMSTAR-2 (A Measurement Tool to Assess Systematic Reviews, version 2).

**Results:**

We initially identified 1004 studies, but only 5 (0.5%) systematic reviews met the inclusion criteria. Email, vodcasts, smartphone apps, and SMS text messaging were the principal telemedicine channels. Telemedicine interventions were safe, helpful, and motivating; improved retention rates and patient-physician communication; and reduced ED symptoms.

**Conclusions:**

Telemedicine interventions showed promising, positive findings as a complementary tool for face-to-face ED treatment that must be interpreted cautiously. The limited number of systematic reviews selected and their moderate to critically low quality underscore the need for further research in this area.

## Introduction

### Background

Eating disorders (EDs) are psychiatric disorders defined as abnormal eating habits that negatively affect a person’s physical or mental health and usually begin in late childhood or early adulthood. These are associated with significant medical, psychiatric, and psychosocial consequences.

The global burden of EDs was estimated at 6.6 million people by 2019 [1]. In Spain, the estimated prevalence was 13% in both sexes [2]. However, the COVID-19 pandemic had a negative impact on EDs, increasing the incidence among young people and the demand for care in most countries [3,4].

The most common EDs include anorexia nervosa (AN), bulimia nervosa (BN), binge eating disorder (BED), and eating disorder not otherwise specified (EDNOS). AN is characterized by an intense fear of gaining weight, persistent behaviors that interfere with weight gain, and a distorted perception of one’s own body image. It often results in dangerously low body weight and can lead to severe medical complications, such as bradycardia, osteoporosis, amenorrhea, and electrolyte imbalances. BN involves recurrent episodes of binge eating—consuming unusually large amounts of food within a discrete period—followed by inappropriate compensatory behaviors, such as self-induced vomiting, excessive exercise, fasting, or misuse of laxatives or diuretics. Unlike AN, individuals with BN typically maintain their body weight within or above the normal range, which can delay recognition and diagnosis. BED is defined as repeated episodes of uncontrollable overeating without the regular use of compensatory behaviors, often accompanied by feelings of guilt, shame, and emotional distress. BED is the most prevalent ED and is frequently associated with overweight and obesity. EDNOS, now referred to in *Diagnostic and Statistical Manual of Mental Disorders, Fifth Edition* as other specified feeding or eating disorder, encompasses clinically significant eating disturbances that do not meet the full criteria for AN, BN, or BED. Examples include atypical AN (significant weight loss despite normal weight), purging disorder, and night eating syndrome. EDNOS represents a substantial portion of ED diagnoses, especially in clinical settings, and is associated with comparable levels of psychological distress and functional impairment [5,6]. The onset of EDs generally occurs between the ages of 10 and 20 years, although BED and EDNOS may appear in slightly older age groups [6].

EDs are associated with numerous physical problems (eg, diabetes, hypertension, and ulcers) and mental disorders (eg, anxiety disorder and depression) [5]. Patients with EDs have significantly higher mortality and suicide rates compared to the standard population [6,7]. The etiopathogenesis of ED is complex and not yet well understood. It may arise from the interaction of multiple risk factors, including genetic, neurobiological, psychological (eg, body image disturbances and personality disorders), developmental factors (eg, childhood sexual abuse), and sociocultural factors [8].

Given that EDs show great variability in their manifestation and severity, the treatment requires multidisciplinary collaboration of psychiatrists, psychologists, general practitioners, nutritionists (or endocrinologists), dieticians, nurses, and occupational therapists [2]. The treatments include medical nutritional support, pharmacological treatments, and psychological therapies. Cognitive behavioral therapies (CBTs) have been shown to be effective for the treatment of EDs and are considered the therapies of choice by psychologists for both treatment and ongoing support of patients with EDs [2]. Many therapists have observed telemedicine as an alternative to face-to-face therapy in extreme health situations, such as during the COVID-19 pandemic, although the availability of technological equipment for its administration has been uneven.

Telemedicine is the use of telecommunication systems to provide health care at a distance [9]. It involves the secure transmission of medical data and information via text, sound, images, or other forms necessary for the prevention, diagnosis, treatment, and monitoring of patients (EU Commission definition, COM {2008}689) [10]. Methods may vary in terms of the way the intervention is technically implemented, using tablets, smartphones, and computers, and the channels such as smartphone apps, websites, SMS text messaging, and vodcast, among others. In recent years, considerable progress has been made in the development and validation of psychological treatments administered through telecommunication systems. The popularity of telemedicine has increased rapidly in recent decades, and researchers have realized its potential in mental health and health in general. Telemedicine is a current trend as it is estimated that more than 50% of the population in high- and middle-income countries suffer from at least one mental disorder in their lifetime, with a significant impact on their quality of life [11].

Several factors have contributed to the growth in the adoption of telemedicine in health care, such as significant advances in communication technologies, the widespread use of high-speed internet, and the COVID-19 pandemic. The COVID-19 pandemic catalyzed the rapid and global adoption of telemedicine solutions in daily practice in all health care fields [12] due to the need for physical distancing and the shortage of health care professionals [13]. However, the implementation of telemedicine also raises technical and privacy concerns, including data security, patient confidentiality, cybersecurity vulnerabilities, and disparities in digital literacy and access to technology. Addressing these issues is crucial to ensuring the safe, ethical, and equitable delivery of care through telecommunication platforms [14-16]. The integration of technology into the study and treatment of EDs has taken many forms, including independent web-based intervention platforms, virtual reality interventions, smartphone apps, and technology-based treatment add-ons [17,18].

### Objectives

We aimed to evaluate the safety and effectiveness of telemedicine as a complementary tool for cognitive face-to-face therapies to promote adherence and monitoring in patients with an ED undergoing treatment.

## Methods

### Overview

We conducted a systematic review of the literature of telemedicine and EDs following the recommendations contained in the PRISMA (Preferred Reporting Items for Systematic Reviews and Meta-Analyses) reporting guidelines [19]. The research question was established in PICO (population, intervention, comparison, and outcomes) format. The review was conducted in accordance with a prespecified protocol, although this was not registered in a public database.

### Source and Search Strategy

We searched the following health evaluation agency websites and databases: the National Institute for Health and Care Excellence, the Canadian Agency for Drugs and Technologies in Health (now known as Canada’s Drug Agency), MEDLINE (Ovid), Embase, Web of Science, Cochrane Library, international HTA database (International Network of Agencies for Health Technology Assessment), CINAHL (EBSCO), and PsycINFO (EBSCO). Structured and systematic search strategies specific to each resource consulted were constructed using free and controlled terminology to identify the relevant studies (Multimedia Appendix 1). The last update was conducted in December 2024 without temporal restrictions.

### Eligibility Criteria

All identified references were initially filtered by title and abstract, and later, after full-text reading, using the following inclusion criteria: systematic reviews, synthesis reports, or meta-analyses studies that address telemedicine as a complementary therapy to face-to-face care; patients with an ED diagnosis and under treatment; and without language limits. Other study designs, editorials, letters to the editor, opinions, notes, surveys, consensus documents, conference abstracts, systematic reviews without assessment of the methodological quality of the included studies, and animal studies were excluded. A review of systematic reviews was selected to avoid redundancy and build on existing high-quality evidence.

### Data Extraction

The authors, country, year of publication, objectives, study design, patient characteristics, databases consulted, date of the search, intervention, time of follow-up, and result measures of the included studies were extracted and summarized in tables.

### Methodological Quality Assessment

Two researchers performed an independent critical reading of the systematic reviews and assessed the risk of bias in the included studies, following the methodology established in AMSTAR-2 (A Measurement Tool to Assess Systematic Reviews, version 2) [20]. Potential disagreements were resolved by consensus.

### Analysis and Synthesis of Results

We performed a qualitative synthesis of the primary outcome measures following the reporting criteria established by the PRISMA guidelines [19]. We collected them in tables for the descriptive and narrative analysis. Although a meta-analysis was initially considered, the substantial heterogeneity in intervention types and outcome measures across the included studies made this approach inappropriate. A qualitative synthesis was chosen to provide a comprehensive overview of the available evidence.

## Results

### Included Studies

**Figure 1:**
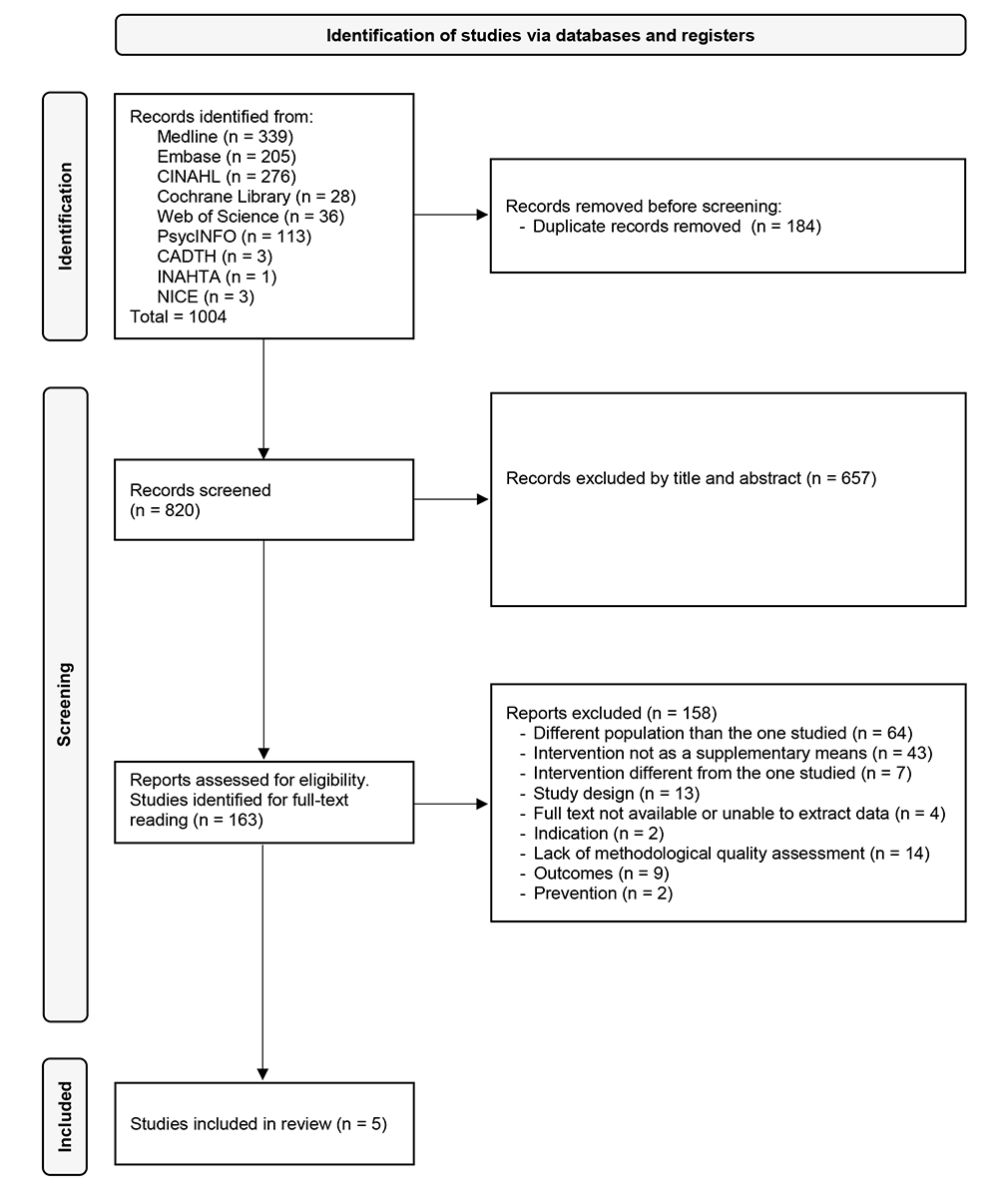
PRISMA (Preferred Reporting Items for Systematic Reviews and Meta-Analyses) flowchart for the study selection. CADTH: Canadian Agency for Drugs and Technologies in Health (now known as Canada's Drug Agency); NICE: National Institute for Health and Care Excellence.

A total of 1004 studies were initially identified from the search in the MEDLINE, Embase, CINAHL, Cochrane Library, Web of Science, PsycINFO, Canadian Agency for Drugs and Technologies in Health (now known as Canada’s Drug Agency) international HTA database, and National Institute for Health and Care Excellence databases and websites, but 184 (18.3%) duplicates were removed before screening. Therefore, 820 studies were included in the screening process. Figure 1 shows a flow diagram of the screening process and the reasons for exclusion. Briefly, 80.1% (657/820) of studies were excluded by title or abstract because they did not meet the inclusion criteria. The remaining 19.9% (163/820) studies were assessed for eligibility. After full-text reading, 96.9% (158/163) studies were excluded due to different reasons, such as different populations than the one studied, intervention not as a supplementary means, or lack of methodological quality assessment, among others. Finally, 5 studies were included in the review.

### Characteristics of the Included Studies

We identified 5 systematic reviews on telemedicine as a complementary tool to face-to-face care, published between 2011 and 2021. The characteristics of these systematic reviews are summarized in Table 1. These reviews varied in their intervention types, study populations, study designs, and geographic focus, reflecting the diverse landscape of telemedicine research in EDs.

**Table 1:** Characteristics of the selected systematic reviews.

Study and year	Intervention	Country	Population	Number of primary studies	Risk of bias assessment	Objective of the study	Resources consulted and search date
Aardoom et al [21], 2013	Email as a therapeutic complement to face-to-face therapy with elements of CBT^a^ (the emails were mostly supportive, including words of encouragement)	United States	Women with AN^b^ and EDNOS^c^; aged 13 to 50 years; women (N=7)	2 observational case series [22,23]	The Cochrane Risk of Bias tool for RCTs^d^. Studies were classified into levels of evidence: the lower the level assigned, the higher the methodological quality	Review the literature on the treatment of ED^e^ via the internet.	MEDLINE, Embase, PsycINFO, and Web of Science; January 2013
Anastasiadou et al [14], 2018	mHealth interventions as an adjunct to face-to-face CBT-based treatment; vodcast intervention with mobile technology; smartphone app; SMS text messages	United Kingdom, United States, and Germany	AN, BN^f^, BED^g^, EDNOS; aged 16 to 67 years; N=351	9 total studies: 2 experimental non-RCT [24,25]; 3 observational case series [26-28]; 1 experimental case series [29]; 2 experimental RCT^j^ [30,31]; and 1 experimental crossover study [32]	Studies were classified into levels of evidence according to the Centre for Evidence-Based Medicine	Systematically review existing evidence for mHealth tools for the treatment of ED.	PubMed, PsycINFO, Scopus, and ClinicalTrials.gov; October 2017
Hay and Claudino [33], 2015	SMS text messages versus usual CBT treatment after hospital discharge (the SMS intervention consisted of a weekly interaction); Email as a therapeutic complement to face-to-face therapy	N/A^h^	Women with BN or EDNOS (N=165); aged >18 years	1 experimental RCT [31] and 1 systematic review [21]	GRADE^i^ system	To assess the effects of online interventions for people with BN.	MEDLINE, Embase, Cochrane Library, DARE^j^, and HTA database; April 2014
Martin et al [16], 2011	Email as a therapeutic complement to face-to-face therapy with elements of CBT	United States	Women with AN and EDNOS; aged 13 to 50 years; female (N=7)	2 observational case series [22,23]	MMAT^k^	To assess the effectiveness and impact of network communication interventions in young people with mental health problems.	MEDLINE, Embase, ASSIA^l^, Sociological Abstracts, Social Studies abstract, PsychINFO, Cochrane Database of Systematic Reviews, dissertation abstracts, and current controlled trials; May 2009
Neumayr et al [34], 2021	Smartphone app as a complement to face-to-face CBT-based therapy	Germany	Women with BN and BED (N=66)	1 experimental RCT [27]	The quality assessment of all included publications was conducted according to 10 of the 14 codes for weaknesses [20]	To identify the apps for the mental disorders depression, anxiety, and ED that have already been evaluated internationally; check their availability in the German app stores, as well as their availability in German; and present the results of the RCTs.	PubMed, PsychINFO, and PSYNDEX; June 2018

^a^CBT: cognitive behavioral therapy.

^b^AN: anorexia nervosa.

^c^EDNOS: eating disorder not otherwise specified.

^d^RCT: randomized controlled trial.

^e^ED: eating disorder.

^f^BN: bulimia nervosa.

^g^BED: binge eating disorder.

^h^N/A: not applicable.

^i^GRADE: Grading of Recommendations, Assessment, Development, and Evaluation.

^j^DARE: Database of Abstracts of Reviews of Effects.

^k^MMAT: Mixed Methods Appraisal Tool.

^l^ASSIA: Applied Social Sciences Index and Abstracts.

The included reviews covered a wide range of telemedicine interventions, including email support (Aardoom et al [21] and Martin et al [16]), smartphone apps (Neumayr et al [34]), SMS text messages (Hay and Claudino [33]), and mHealth tools such as vodcasts (Anastasiadou et al [14]). These interventions were generally designed as adjuncts to face-to-face CBT, with a focus on providing motivational support, symptom monitoring, and psychoeducation.

The studies included in these reviews targeted various populations with EDs, including individuals with AN, BN, BED, and EDNOS. Most of the included studies focused primarily on women (513/617, 86.5%), although 2 reviews (Anastasiadou et al [14] and Neumayr et al [34]) included studies with both male and female participants, representing 13.52% of the total study population. This gender imbalance reflects the typical distribution of EDs but highlights the need for more inclusive research in this area.

The included systematic reviews incorporated a variety of study designs, including randomized controlled trials (RCTs), non-RCT experimental studies, observational case series, and crossover studies. Notably, there was some overlap in the primary studies included in these reviews. For example, the RCT by Hildebrandt et al [30] was included in the studies by both Anastasiadou et al [14] and Neumayr et al [34], while the observational case series studies by Yager et al [22,23] were included in the studies by both Martin et al [16] and Aardoom et al [21].

As summarized in Table 1, most of the included studies were conducted in high-income countries, including the United States, the United Kingdom, and Germany. This geographic concentration may limit the generalizability of the findings, as telemedicine interventions can vary significantly in their accessibility and effectiveness across different health care systems and socioeconomic contexts.

Several primary studies were included in the multiple reviews, highlighting the interconnected nature of the evidence base in this field. For example, the case series included in the reviews by Martin et al [16] and Aardoom et al [21] were largely overlapping, as were some of the experimental studies included in the reviews by Anastasiadou et al [14] and Hay and Claudino [33]. This overlap underscores the importance of carefully evaluating the quality and relevance of included studies to avoid double-counting evidence in future reviews.

Overall, the included reviews reflect a diverse but sometimes overlapping body of evidence on the use of telemedicine as a complementary tool for the treatment of EDs, emphasizing the need for more rigorous primary studies with broader population samples and more standardized outcome measures.

### Quality Assessment

None of the 5 systematic reviews performed a meta-analysis. The methodological quality of the reviews was evaluated by applying AMSTAR-2 domains criteria [20] (Multimedia Appendix 2 [14,16,20,21,33,34]).

The systematic reviews by Martin et al [16] and Hay and Claudino [33] showed a moderate level of confidence. The main weaknesses were that they did not explicitly state that the review methods were established before the conduct of the review so that any significant deviation from the protocol could be justified [16], the exhaustive literature search strategy was partially presented [16,33], and their failure to explicitly provide the list of excluded studies and the justification for their exclusions [16,33]. Other methodological weaknesses found were related to the absence of information on the decision to include study design in the reviews analyzed [16,33], the failure to conduct peer selection or data extraction [16,33], and the absence of information on aspects related to the funding of the included studies 15,20]. The systematic reviews by Anastasiadou et al [14] and Neumayr et al [34] showed a low level of confidence. The main weaknesses were that they did not explicitly state that the review methods were established before the conduct of the review so that any significant deviation from the protocol could be justified [14,34], they partially presented the exhaustive literature search strategy [14,34], their failure to explicitly provide the list of excluded studies and the justification for their exclusions [14,34], and they did not explicitly provide the observed heterogeneity in the results of the review [14,34]. Other limitations found in these reviews were related to the absence of information on the decision to include the study design in the reviews analyzed and the absence of information on aspects related to the funding of the included studies. Finally, the systematic review by Aardoom et al [21] demonstrated a critically low level of confidence. The critical weaknesses were the failure to explicitly state that the review methods were established before the conduct of the review, making it difficult to justify any significant deviations from the protocol, as well as the absence of a predefined protocol. In addition, there was a partial lack of an exhaustive literature search strategy and a missing list of excluded studies, along with the reasons for their exclusion. Other identified limitations involved the lack of information regarding the decision to include specific study designs, the absence of peer selection or data extraction, and missing details about the funding of the included studies.

### Safety of Telemedicine Interventions

The systematic reviews did not find direct adverse effects of telemedicine interventions on patients’ health. However, Martin et al [16] and Anastasiadou et al [14] identified some patients’ concerns about computer privacy using email and smartphone apps, respectively; particularly, with the personalized nature of SMS text messages treatment of patients with AN and BN (Multimedia Appendix 3 [14,16,21]).

The next section first describes the modalities of the technological interventions and then presents them organized by the conditions addressed.

### Efficacy and Effectiveness of Each Telemedicine Intervention

#### Email

The systematic reviews by Aardoom et al [21] and Martin et al [16] included the same 2 observational case series studies [22,23] assessing the email as a complementary tool for the anorexia and EDNOS treatments (Tables 2 and 3). These studies were classified as having a low level of quality, and qualitative data could not be extracted. Patients considered the email helpful, motivating, and a good approach to keep in touch with the therapist, as it freed up time in face-to-face sessions. Patients highlighted that email provided positive emotional warmth because they could communicate with therapists whenever needed (Multimedia Appendix 3).

**Table 2:** Characteristics of the included studies with telemedicine intervention as a complementary tool of face-to-face therapy in patients with anorexia nervosa (AN; level 1: double-blind randomized trials, level 2: open randomized trials, level 3: observational studies, level 4: small observational studies, and level 5: case series, case reports, and expert opinions).

Study, intervention, and comparator				Follow-up		Number of primary studies		Results		Quality of studies
**Aardoom et al [21], 2013**										
	**Email as a complement**									
		No comparator	No follow-up		1 observational case series [22]		N/A^a^		Level 5	
**Anastasiadou et al [14], 2018**										
	**Vodcast: “supported eating” intervention with mobile technology (vodcast video with auditory and visual images of the ED^b^)**									
		CG^c^: music	Before and after 20-min feeding test.		1 non-RCT^d^ experimental study [25]		Primary outcome: amount of smoothie drunk: vodcast: mean 139.2, SD 103.6; music: mean 124.6, SD 105.3; t17=2.2; P<.05 Secondary outcome: anxiety and positive mood (VASe): AN anxiety—t17=2.2; P<.05 (IGf: mean change 1.7, SD 2.2; CG: mean change 0.4, SD 2.5). AN negative thought—t17=2.1; P<.05 (IG: mean change 1.5, SD 2.2; CG: mean change 0.0, SD 2.4). AN positive mood—t17=−2.2; P<.05 (IG: mean change −1.4, SD 1.7; CG: mean change −0.5, SD 2.3)		Level 2	
	
		Inpatient and outpatient CG: music	Before and after 20-min feeding test.		1 non-RCT experimental study [24]		Primary outcome: desire to eat: hospitalized patients— vodcast: mean change −1.7, SD 2.8 versus music: mean change −1.3, SD 1.9; t18=2.7; P=.01. Outpatients—vodcast: mean change −0.9, SD 2.2 versus music: mean change −0.7, SD 1.6. Secondary outcome: attentional bias to food (VAS). Hospitalized patients—vodcast: mean change 26.5, SD 61.7 versus music: mean change 21.0, SD 53.4; t18=−2.5; P=.02; ESg=0.8. Outpatients—vodcast: mean change −9.7, SD 31.8 versus music: mean change −4.8, SD 33.5; ES=0.3.		Level 2	
	**Vodcasts: energy controller, mindful eating, and motivational reflection (participants received a DVD with 3 vodcasts for use at home)**									
		No comparator	Follow-up 1 to 2 weeks Follow-up 2 to 3 months		1 observational case series [26]		Primary outcome: the amount of shake consumed; food-related anxiety—N/A^g^		Level 4	
**Martin et al [16]**										
	**Email as a complement.**									
		No comparator	No follow-up		1 observational case series [22]		N/A		MMAT^h^: 1.1^i^ yes; 1.2 yes; 1.3 yes; 1.4 yes; 1.5 N/A; 1.6 yes	

^a^N/A: not applicable.

^b^ED: eating disorder.

^c^CG: control group.

^d^RCT: randomized controlled trial.

^e^VAS: visual analogue scale.

^f^IG: intervention group.

^g^ES: effect size.

^h^MMAT: Mixed Methods Appraisal Tool.

^i^1.1: Is a qualitative objective or question stated? 1.2: Is an appropriate qualitative approach, design, or method described? 1.3: Is the study context described and findings related to it? 1.4: Are participants described and is sampling justified? 1.5: Are qualitative data collection and analysis processes described? 1.6: Do researchers describe their reflexivity?

**Table 3:** Characteristics of the included studies by telemedicine intervention in patients with heterogeneous disorders (heterogeneous population; level 1: double-blind randomized trials, level 2: open randomized trials, level 3: observational studies, level 4: small observational studies, and level 5: case series, case reports, and expert opinions).

Study, population, and intervention		Comparator	Follow-up	Number of primary studies	Results	Quality of studies
**Aardoom et al [21], 2013**						
	Population: people with AN^a^ and ED^b^; intervention: email	No comparator	No follow-up	1 observational case series [23]	N/A^c^	Level 5
**Anastasiadou et al [14], 2018**						
	Population: people with AN and BN^d^; intervention: smartphone app: self‐monitoring through the “Food for thought” app. (Patients used app [CBT^e^‐based] and clinicians answered questions at any time)	No comparator	8 weeks after initial registration	1 observational case series [27]	N/A	Level 4
	Population: people with BN and BED^f^; intervention: smartphone app: “Noom monitor.” (12 weeks: CBT‐GSH^g^+app)	CBT‐GSH	4, 8, 12, 24, and 36 weeks	1 experimental RCT^h^ [30]	Primary outcome: OBEsi, SBEsj, and compensatory behaviors. OBEs: within‐group effect—positive and significant; between groups effect—positive; within‐group effect size=0.41. SBEs: within‐group effect—positive and significant; between groups effect nsk; within‐group effect size=0.18. EDE-Ql: within‐group effect—positive and significant; between groups effect—ns. BMI: within‐group effect—positive and significant	Level 2
	Population: people with AN and BN; intervention: SMS text messaging (8 weeks: 4 weeks CBT+motivational text messages and 4 weeks without any text messages)	No comparator	8 weeks	Experimental crossover [32]	Primary outcomes: EDE-Q and RMQm. EDE-Q dietary restraint: intragroup effect—positive and significant; between groups effect—N/A; within‐group effect size=2.01. RMQ precontemplation: t12=4.26; P=.001; preintervention mean 76.67, SD 21.88; postintervention mean 37.67, SD 24.44. RMQ action: t12=−6.08; P<.001; preintervention mean 30.42, SD 33.208; postintervention mean 73.75, SD 28.13. RMQ confidence: t12=−2.25; P=.046; preintervention mean 30.42, SD 33.208; postintervention mean 73.75, SD 28.13	Level 2
	Population: people with BN and EDNOS^n^; intervention: SMS text messages versus TAU^o^ after hospital discharge.	TAU	4 months and 8 months	1 RCT [31]	Primary outcome: remission rate. Remission ITTp analysis: IGq=51.2%; CGr=36.1%; χ21=3.81; P=.05. Secondary outcome: abstinence and full symptom rates. Abstinence ITT analysis: IG=37.8%; CG=18.1%; χ21=7.99; P<.01. Full symptom rate ITT analysis: IG=32.9%; CG=54.2%; χ21=7.60; P<.01	Level 2
**Hay and Claudino [33], 2015**						
	Population: people with BN and EDNOS; intervention: SMS text messaging versus usual treatment after hospital discharge (the text messaging intervention consisted of a weekly interaction for 16 weeks after discharge. Participants used their private mobile phones to report body dissatisfaction, frequency of binge eating, and frequency of compensatory behaviors, and the software followed an algorithm to provide a personalized feedback message)	TAU	4 months and 8 months	1 RCT [31]	Intergroup abstinence (absence of binge eating and compensatory behaviors for a minimum of 4 weeks) at 8 months: IG—38% (31/82) with text messaging intervention; CG—18% (15/83); P<.01 Intergroup effect+remission (maximum of 1 binge eating episode per week and a maximum of 1 compensatory behavior per week for no less than 4 weeks) at 8 months: IG—51% (42/82); CG—36% (30/83); P=05 Intergroup effect+full symptoms (still meet DSM-IVs diagnostic criteria for BN or EDNOS) at 8 months: IG—33% (27/82); CG—54% (45/83); P<.01; between groups effect—positive and significant	Very low
**Martin et al [16], 2011**						
	Population: people with AN and ED; intervention: email	No comparator	No follow-up	1 observational case series [23]	N/A	MMAT^t^: 1.1^u^ yes; 1.2 yes; 1.3 N/A; 1.4 N/A; 1.5 yes; 1.6 yes
**Neumayr et al [34], 2021**						
	Population: people with BN and BED; intervention: smartphone app, “Noom monitor” (12 weeks; CBT‐GSH+app)	CBT‐GSH	4, 12, 24, and 36 weeks	1 experimental RCT [30]	Adherence (of full treatment): 81.8% EDE-Q (bulimic and compensatory episodes; 12 weeks; between groups): mean 0.41, 95% CI −0.13 to 0.95	Exclusive presence of self-assessment tools as a measure of results

^a^AN: anorexia nervosa.

^b^ED: eating disorder.

^c^N/A: not applicable.

^d^BN: bulimia nervosa.

^e^CBT: cognitive behavioral therapy.

^f^BED: binge eating disorder.

^g^GSH: guided self-help.

^h^RCT: randomized controlled trial.

^i^OBE: objective bulimic episode.

^j^SBE: subjective bulimic episode.

^k^ns: no significant differences between the groups.

^l^EDE-Q: eating disorder examination questionnaire.

^m^RMQ: Readiness and Motivation Questionnaire.

^n^EDNOS: eating disorder not otherwise specified.

^o^TAU treatment as usual.

^p^ITT: intent to treat.

^q^IG: intervention group.

^r^CG: control group.

sDSM-IV: Diagnostic and Statistical Manual of Mental Disorders, Fourth Edition.

^t^MMAT: Mixed Methods Appraisal Tool.

^u^1.1: Is a qualitative objective or question stated? 1.2: Is an appropriate qualitative approach, design, or method described? 1.3: Is the study context described and findings related to it? 1.4: Are participants described and is sampling justified? 1.5: Are qualitative data collection and analysis processes described? 1.6: Do researchers describe their reflexivity?

#### Vodcast

The systematic review by Anastasiadou et al [14] included 2 non-RCT experimental studies [24,25] and 1 observational case series study [26] that evaluated the vodcast as a complementary tool for ED treatment. The quality levels of these studies were moderate and low, respectively. The non-RCT used the vodcast “Supported eating” by mobile technology, and the observational study used the vodcast “Energy controller, mindful eating, motivation reflection” provided on a DVD. Regarding the non-RCT studies, within-group improvements in symptoms were found to be related to reductions in anxiety (*P*<.05) and an improvement in mood. In addition, an increase in food consumption and a more significant decrease in distress and attentional bias to food were also observed after the vodcast intervention among the patient group [24]. Regarding the observational case series study, the vodcasts were well accepted by the patients, who considered them useful, supportive, and motivating. They observed an increase in food intake and weight gain [26]. However, in the non-RCT [24], this mode of intervention was not considered very useful (Table 2; Multimedia Appendix 3).

#### Smartphone App

The systematic reviews by Anastasiadou et al [14], and Neumayr et al [34] included 1 RCT and 2 observational case studies that assessed smartphone apps; particularly, the Noom monitor app for patients with BN and BED [30], the CBT-based Food for Thought app for patients with AN and BN [27], and a smartphone app with the self-help program with personalized CBT and ecological momentary intervention modules for the treatment of patients with BED [28], respectively. The RCT [30] was included in both systematic reviews. The quality of these studies was moderate to low. According to the RCT study, smartphone app use reduces binge eating and purging episodes and increases patients’ body mass. Regarding the observational case series studies, patients considered the smartphone apps feasible and acceptable and increased the frequency of food recording compared to the traditional method. However, the RCT did not show any differences in adherence and alliance between the intervention and control groups. Patients with BED participating in observational studies disclosed concerns about the personalization and privacy of this method (Tables 2-5-; Multimedia Appendix 3).

#### SMS Text Messages

The systematic reviews by Anastasiadou et al [14] and Hay and Claudino [33] included an experimental case study [29], a crossover experimental study [32], and an RCT [31] evaluating SMS text messaging as a complementary tool for BN, EDNOS, and AN treatment (Tables 2-4). Both systematic reviews included the same RCT [31]. SMS text messaging significantly decreased the number of episodes of purging and binge eating, symptoms of depression and ED, and late-night meals [14]. Similarly, significant differences were observed in remission rates between the intervention and control groups [14,33] (Table 5). The acceptability and feasibility of SMS text messaging by their users were satisfactory [14]. The feasibility scores were 91.5% of the daily entries and 87.4% of the daily food records in the crossover experimental study. Adherence was 87% in the experimental case series. Patients found the reminders helpful despite some doubts about personalizing the messages (Multimedia Appendix 3).

**Table 4:** Characteristics of the included studies with telemedicine intervention as a complementary tool of face-to-face therapy in patients with bulimia nervosa (BN; level 1: double-blind randomized trials, level 2: open randomized trials, level 3: observational studies, level 4: small observational studies, and level 5: case series, case reports, and expert opinions).

Study and year	Intervention	Comparator	Follow-up	Number of primary studies	Results	Quality of study
Anastasiadou et al [14], 2018	SMS text‐ messaging: program for self‐ monitoring BN symptoms with CBT^a^ components	No comparator	12 weeks and 24 weeks	1 experimental case series [29]	Primary outcome: BDI^b^; EDI^c^; NEQ^d^ (number of binges and number of purges). BDI: t_15_=X; *P*<.001; baseline—mean 23.1, SD 0.7; 12 weeks—mean 11.4, SD 9.6; 24 weeks—mean 8.8, SD 9.4. EDI: within‐group effect—positive and significant; within‐group effect size=1.26. NEQ: within‐group effect—positive and significant; within‐group effect size=0.61. Binges past week: within‐group effect—positive and significant; within‐group effect size=0.90. Purges past week: within‐group effect—positive and significant; within‐group effect size=0.72	Level 4

^a^CBT: cognitive behavioral therapy.

^b^BDI: Beck Depression Inventory.

^c^EDI: Eating Disorder Inventory questionnaire.

^d^NEQ: Night Eating Questionnaire.

**Table 5:** Characteristics of the included studies with telemedicine intervention as a complementary tool of face-to-face therapy in patients with binge eating disorder (BED; level 4: small observational studies).

Study, year	Intervention	Comparator	Follow-up	Number of primary studies	Results	Quality of study
Anastasiadou et al [14], 2018	Smartphone app: self‐help program for BED; IG^a^: patients used a self‐help program with CBT^b^ modules+personalized EMI^c^+self-monitoring	No comparator	Not reported	1 observational case series [28]	N/A^d^	Level 4

^a^IG: intervention group.

^b^CBT: cognitive behavioral therapy.

^c^EMI: ecological momentary intervention.

^d^N/A: not applicable.

### Efficacy and Effectiveness of Telemedicine Intervention by Eating Disorder

#### Anorexia Nervosa

Two non-RCT experimental studies [24,25] included in the systematic review by Anastasiadou et al [14] showed that vodcasts as a complementary treatment significantly reduced anxiety (*P*<.05) and negative thoughts (*P*<.05) and positively improved the mood of the intervention group participants in the subsequent evaluations compared to the control group receiving music treatment. In addition, vodcast increased food consumption and significantly decreased the distress and attentional bias to food after the intervention [14]. However, regarding the usefulness of vodcasts, one of the non-RCT experimental studies included in this review considered it not very useful and too focused on ED problems. In contrast, the observational study included in the same review highlighted that vodcast use increased food intake, reduced anxiety, and increased weight after 3 months. In this observational study, vodcasts were considered useful and a source of support and motivation.

According to a case series study included in the systematic reviews by both Aardoom et al [21] and Martin et al [16], email was a useful complementary therapy because it allowed patients to express themselves more easily and freed up time in face-to-face sessions in an outpatient setting. Table 2 and Multimedia Appendix 3 provide detailed information.

#### Bulimia Nervosa

The systematic review by Anastasiadou et al [14] included an experimental case series [29] assessing the within-group effect of SMS text messaging for self‐monitoring BN symptoms with CBT components after 12- and 24 weeks from intervention. Binge eating and purging episodes decreased significantly (within‐group effect size was 0.90 and 0.72, respectively). Symptoms of depression (Beck Depression Inventory: *P*<.001), ED (Eating Disorder Inventory: effect size=1.26), and nighttime eating (Night Eating Questionnaire: effect size=0.61) decreased significantly. In addition, the acceptability was above average, and the adherence was 87%. The attrition was 48.4% of the total study population and 60% of those who started treatment. Table 4 and Multimedia Appendix 3 provide detailed information.

#### Binge Eating Disorder

The systematic review by Anastasiadou et al [14] included an observational case series [29] assessing the use of a smartphone app with a self-help program that includes modules on CBT, personalized ecological momentary intervention, and self-monitoring. However, no information is available on its effects (Table 5). Patients considered the intervention feasible and acceptable. However, they expressed concerns about the personalization and adaptability of the app, as well as privacy and sharing issues (Multimedia Appendix 3).

#### Heterogeneous Study Population

Most of the primary studies included in the 5 systematic reviews were performed on a heterogeneous population of patients presenting different EDs. Table 3 and Multimedia Appendix 3 summarize the telemedicine effect found and the qualitative information extracted from these studies containing a heterogeneous population, respectively.

Briefly, the systematic reviews by Anastasiadou et al [14] and Neumayr et al [34] included an RCT [30] evaluating the Noom monitor app as a complementary tool for the treatment of patients with BN and BED. The study revealed greater reductions in binge eating and purging episodes than in the control group (objective bulimic episodes intergroup effect=0.41) and within-group improvements in ED-related symptoms (objective bulimic episodes, subjective bulimic episodes, eating disorder examination questionnaire) and the increase in BMI. No differences in treatment adherence or adherence were reported when compared to the control group.

The systematic reviews by Anastasiadou et al [14] and Hay and Claudino [33] included an RCT [31] evaluating the SMS text messages to prevent relapses in patients with BN and EDNOS. The study showed that SMS improved the abstinence levels at 8 months (37.8% vs 18.1%; *P*<.01), reduced the rate of women having complete symptoms of BN and EDNOS (32.9% vs 54.2%; *P*<.01), and increased remission rates (51.2% vs 35.1%; *P*=.05) compared to the usual treatment. In addition, the study by Anastasiadou et al [14] included an experimental crossover study [32] assessing SMS text messages to prevent relapse in patients with AN and BN. In this experimental crossover study, patients significantly reduced dietary restriction at 8 weeks (in-group effect size based on eating disorder examination questionnaire score=2.01) and significantly increased their willingness to change as measured by the Readiness and Motivation Questionnaire (*P*<.05). SMS text messaging was considered a feasible complementary tool (based on the 91.5% of the daily entries and 87.4% of the daily food records), with satisfactory acceptability (mean 7.05, SD 2.36 out of 10 points) and high retention rate (92.2%). Overall, users liked the text messages and the reminders’ usefulness, although some disclosed dissatisfaction with the personalized nature of the messages.

Finally, the systematic reviews by Aardoom et al [21], Anastasiadou et al [14], and Martin et al [16] included observational studies assessing email and the Food for thought smartphone app as complementary tools for the treatment patients with AN, BN, and ED. These studies highlighted that email increased the frequency and time of contact between patients and clinicians, and that the Food for thought app increased the frequency of food registration compared to pen and paper.

### Synthesis of Findings

Table 6 synthesizes the main findings extracted from the 5 systemic reviews selected, presented by type of ED, study design, and telemedicine intervention.

**Table 6:** Summary of findings.

ED^a^ and the number of primary studies			Telemedicine resource		Summary of results
**Anorexia nervosa [14,16,21]**					
	2 non-RCT^a^ experimental studies [24,25]	Vodcast		Significant increase in the amount of food consumed Significant reduction in anxiety and negative thoughts Significant improvement in positive mood Significant decrease in distress and attentional bias to food Considered unhelpful and too focused on eating disorder issues	
	2 observational case series [23,26]	Vodcast		Increased food intake Reduction in anxiety Weight gain Considered useful as a source of support and motivation	
	2 observational case series	Email		Email use is experienced as positively useful Frees up time in face-to-face sessions and allows patients to express themselves more easily Concern about unwanted disclosures to third parties stemming from a lack of computer privacy	
**Bulimia [14]**					
	1 experimental case series [29]	SMS text messages		Significant decrease in the number of binge eating and purging episodes (within-group) Significant decrease in symptoms of depression and EDIc (within the group) Significant decrease in late-night meals (within-group) Above-average acceptability 87% of adherence	
**BED^d^ [14]**					
	1 observational case series [28]	Smartphone app		Feasible and acceptable intervention Concern for customization and adaptability of the app Concerns about privacy and sharing	
**Heterogeneous study population [14,16,21,33,34]**					
	1 experimental RCT (BN^e^ and BED) [30]	Smartphone app		Significant reductions in binge eating and purging episodes Significant within-group improvements in ED-related symptoms Increased BMI	
	1 experimental RCT (BN and EDNOS^f^) [31]	SMS text messages		Significant improvement in abstinence Significant reduction in the proportion of women who continued to have full symptoms Significant increase in retention rate	
	1 observational case series (AN^g^ and ED) [23]	Email		Email use is experienced positively Helpful and motivating A good approach to keep in touch with their therapist and make them aware of their eating behaviors and problems Lack of satisfaction with the personalized nature of messages	
	1 observational case series (AN and BN) [28]	Smartphone app		Increased frequency of food recording Wide range of use Acceptable and easy to use A broad spectrum of patients	
	1 experimental crossover study (AN and BN) [32]	SMS text messages		Significant reduction in dietary restriction Significant increase in willingness to change Considered feasible Satisfactory acceptability High retention rate Reminders were considered useful Some participants in this study were not satisfied with the personalized nature of the messages	

^a^ED: eating disorder.

^b^RCT: randomized controlled trial.

^c^EDI: Eating Disorder Inventory questionnaire.

^d^BED: binge eating disorder.

^e^BN: bulimia nervosa.

^f^EDNOS: eating disorder not otherwise specified.

^g^AN: anorexia nervosa.

## Discussion

### Principal Findings

Despite the global burden of EDs [1,3,4] and the rapid and global adoption of telemedicine solutions in health care [12], only 5 systematic reviews were identified that discuss telemedicine interventions as a complementary tool to face-to-face ED treatments [14,16,21,33,34]. These reviews encompass a high heterogeneity of primary studies, highlighting the need for more comprehensive research in this area.

Regarding the effectiveness of these interventions, promising outcomes were observed, as presented in Textbox 1.

**Effective outcomes of telemedicine interventions.**

**Anorexia nervosa**

Vodcast as a complementary treatment for anorexia nervosa (AN) was associated with substantial reductions in anxiety, negative thoughts, and improved positive mood compared to the control group. It also led to increased food consumption and reduced anxiety levels.

**Bulimia nervosa**

Significant reductions in binge eating, purging episodes, symptoms of depression, and nighttime eating were observed.

**Binge eating disorder**

In the observational study using a smartphone app, patients found the intervention feasible and acceptable.

**Mixed eating disorders**

Use of smartphone apps in patients with bulimia nervosa (BN) or binge eating disorder significantly reduced binge eating and purging episodes and improved both objective and subjective bulimic episodes within the group. It also increased BMI in these populations. SMS text messaging significantly reduced dietary restriction, improved readiness and motivation (as measured by the Readiness and Motivation Questionnaire), and reduced overall symptom severity in patients with AN or BN.

This study identified email, vodcasts, smartphone apps and SMS text messaging as the principal telemedicine channels offering patients access and adherence to treatment, improving patient-physician communication, and increasing patients’ motivation. In most cases, patients highlighted the usefulness of telemedicine interventions. However, security and privacy issues remain a challenge, as some patients expressed concerns about the use of email and smartphone apps. Patients with EDs, who often struggle with distorted self-perception, may be particularly sensitive to privacy concerns [14].

Although none of the systematic reviews identified direct adverse effects of telemedicine interventions on patients’ health, concerns about privacy in the use of email and smartphone apps were noted. Furthermore, while no technical or organizational problems were explicitly reported in the studies, the scientific literature highlights potential issues, such as technical problems [14], sound and video quality [16], difficulties in ensuring privacy (of parents and children), lack of adequate devices for telemedicine access, and connectivity problems [15]. These factors could impact the effectiveness and safety of telemedicine interventions.

No direct adverse effects of telemedicine intervention on patients’ health were disclosed, although concerns about privacy in the use of email and smartphone apps were identified in some patients [14,16].

Telemedicine intervention showed promise as a complementary tool for face-to-face ED treatment with potential benefits for both patients and health care systems. However, it is important to acknowledge that most primary studies included in the systematic reviews were observational case series and non-RCTs, which limited the quantitative data on effectiveness. This highlights the urgent need for further high-quality research in this field. The systematic review by Anastasiadou et al [14] highlighted the RCT study by Hildebrandt et al [30], which found that smartphone apps as an adjunct ED therapy in heterogeneous study populations increased BMI. These findings are particularly significant for patients with BN and BED, who are often overweight.

In terms of treatment adherence, some primary studies revealed a promising 87% retention rate among patients with BN who used SMS text messaging [29] and 78.8% among patients with BN and BED who used the Noom monitor app [30]. However, it is important to note that the potential efficacy of telemedicine in promoting treatment adherence is an area that requires more research, as the RCT [30] did not find differences in patients with BN and BED using the smartphone app compared with the control group.

The methodological quality of systematic reviews revised in this study ranged from moderate to critically low. This variation in confidence levels could potentially impact the risks of bias in their results. However, it is important to note that the AMSTAR-2 tool, which we used for this evaluation, does not consider the quality of the studies included in each document. Most of the primary studies included in the selected systematic reviews were observational studies with a small number of patients, leading the authors of the systematic reviews to classify them as low quality. We identified only 2 RCTs and 3 experimental studies among the 5 systematic reviews. It is worth mentioning that 2 case series and 2 RCTs were duplicated in some of the identified systematic reviews. The primary studies also had a limited number of interventions for the control group.

Given the limited literature specifically focused on EDs, it is also important to consider the findings of research on other mental health conditions. EDs frequently co-occur with psychiatric diagnoses, such as anxiety disorders, major depressive disorder, and obsessive-compulsive disorder, and share overlapping risk factors and symptomatology. Evidence from studies on these conditions has shown that telemedicine can be effective in supporting symptom monitoring, improving treatment engagement, and facilitating access to care, particularly in underserved populations. These broader findings may offer valuable insights into the potential benefits and limitations of telemedicine-based interventions for individuals with EDs, especially when comorbidities are present. Future studies on EDs should take this into account and incorporate more integrated approaches that address both EDs and commonly associated mental health disorders.

### Limitations

The main limitation of our study is the limited number of systematic reviews identified that analyzed the effects of telemedicine interventions on face-to-face therapies in patients with ED. Overall, these systematic reviews showed a wide heterogeneity in the study designs, telemedicine interventions, ED conditions in the study population, follow-up periods, and outcome measures included in their selected primary studies. This heterogeneity undermines the quality level of of confidence and reduces the robustness of efficacy conclusions.

The studies included in this review were primarily conducted in high-income countries, such as the United Kingdom, the United States, and Germany. This geographical concentration likely reflects where telemedicine infrastructure is more established, funding for mental health research is more readily available, and digital health interventions are more commonly integrated into clinical practice. As a result, the findings may not be fully generalizable to low- and middle-income countries, where access to technology, internet connectivity, and specialized care for eating disorders remains limited. Socioeconomic disparities must therefore be considered when interpreting the applicability of telemedicine as a complementary approach in more diverse global settings.

### Knowledge Gaps and Future Research

The heterogeneity of telemedicine intervention channels and ED conditions in the study population challenges the establishment of robust studies. More RCTs are needed to evaluate the usefulness and clinical efficacy of telemedicine interventions as complementary tools for face-to-face therapies in patients with EDs. Recording data based on established tools, such as assessment tools in EDs [35], may allow better analysis and comparison of the results.

Although few studies have evaluated telemedicine as a complementary treatment to face-to-face therapies, existing evidence suggests that this approach is promising for the treatment of EDs in youth. Previous studies have shown that telemedicine, particularly internet-based interventions such as internet-based CBT, can significantly improve pathological eating behaviors and reduce binge episode frequency in patients with binge-spectrum eating disorders [36,37]. Moreover, while further research is needed, especially regarding the use of videoconferencing and mobile apps, current findings support its potential as an effective tool within the therapeutic framework [38-41].

Although the reviewed studies suggest that telemedicine can improve treatment adherence and patient-clinician communication, evidence on its long-term impact on recovery and relapse prevention is limited. Studies with extended follow-up periods are needed to assess whether the short-term benefits persist over time.

### Conclusions

In our review, 5 systematic reviews were identified that reported telemedicine as a complementary treatment to face-to-face therapies. The telemedicine tools identified in the included studies were vodcasts, SMS text messaging, smartphone apps, and email.

The synthesis of the results was carried out qualitatively due to the significant heterogeneity of the interventions, the population included, and different outcome measures and follow-up periods. It should be noted that the studies included in the reviews identified for analysis were of various designs and that the quality of the evidence reviewed was low or moderate. Tables 2-6 provide a detailed summary of the results and the quality of the included studies that can be used for the correct interpretation of the following conclusions:

- No direct adverse effects of telemedicine on patient health were identified, although concerns regarding privacy in the use of email and smartphone apps remain a significant consideration for future research.
- Telemedicine interventions, including vodcasts and smartphone apps, have shown promise in improving patient motivation, reducing negative thoughts, and increasing adherence to treatment in patients with various EDs.
- Smartphone apps and SMS text messaging have been associated with reductions in binge eating, purging episodes, and dietary restrictions, as well as improvements in readiness to change and overall symptom reduction.

Despite these potential benefits, the overall quality of the evidence remains limited, with most included studies being observational case series with small sample sizes, leading to moderate to low confidence in the overall findings. Further high-quality research is needed to confirm the effectiveness of telemedicine as a complementary treatment for EDs.

## Appendix

Multimedia Appendix 1 Search strategies.

Multimedia Appendix 2 Quality assessment.

Multimedia Appendix 3 Qualitative findings Table A3.

Multimedia Appendix 4 PRISMA (Preferred Reporting Items for Systematic Reviews and Meta-Analyses) checklist.

## Abbreviations

AMSTAR-2: A Measurement Tool to Assess Systematic Reviews, version 2
AN: anorexia nervosa
BED: binge eating disorder
BN: bulimia nervosa
CBT: cognitive behavioral therapy
EDs: eating disorders
EDNOS: eating disorder not otherwise specified
RCT: randomized controlled trial

## References

[1] Santomauro DF, Melen S, Mitchison D, Vos T, Whiteford H, Ferrari AJ (2021). The hidden burden of eating disorders: an extension of estimates from the Global Burden of Disease Study 2019. Lancet Psychiatry.

[2] Gómez Candela C, Palma Milla S, Miján-de-la-Torre A, Rodríguez Ortega P, Matía Martín P, Loria Kohen V, Campos Del Portillo R, Virgili Casas MN, Martínez Olmos MÁ, Mories Álvarez MT, Castro Alija MJ, Martín-Palmero Á (2018). Consenso sobre la evaluación y el tratamiento nutricional de los trastornos de la conducta alimentaria: anorexia nerviosa, bulimia nerviosa, trastorno por atracón y otros. Nutr Hosp.

[3] Taquet M, Geddes JR, Luciano S, Harrison PJ (2021). Incidence and outcomes of eating disorders during the COVID-19 pandemic. Br J Psychiatry.

[4] Solmi F, Downs JL, Nicholls DE (2021). COVID-19 and eating disorders in young people. Lancet Child Adolesc Health.

[5] Button EJ, Chadalavada B, Palmer RL (2010). Mortality and predictors of death in a cohort of patients presenting to an eating disorders service. Int J Eat Disord.

[6] Udo T, Bitley S, Grilo CM (2019). Suicide attempts in US adults with lifetime DSM-5 eating disorders. BMC Med.

[7] Himmerich H, Hotopf M, Shetty H, Schmidt U, Treasure J, Hayes RD, Stewart R, Chang C (2019). Psychiatric comorbidity as a risk factor for mortality in people with anorexia nervosa. Eur Arch Psychiatry Clin Neurosci.

[8] Rikani AA, Choudhry Z, Choudhry AM, Ikram H, Asghar MW, Kajal D, Waheed A, Mobassarah NJ (2013). A critique of the literature on etiology of eating disorders. Ann Neurosci.

[9] Ryu S (2012). Telemedicine: opportunities and developments in member states: report on the second global survey on eHealth 2009 (Global Observatory for eHealth Series, volume 2). Healthc Inform Res.

[10] Communication from the Commission to the European Parliament, the Council, the European Economic and Social Committee and the Committee of the Regions on telemedicine for the benefit of patients, healthcare systems and society. EUR-Lex Access to European Union Law.

[11] Trautmann S, Rehm J, Wittchen H (2016). The economic costs of mental disorders: do our societies react appropriately to the burden of mental disorders?. EMBO Rep.

[12] Seivert S, Badowski ME (2021). The rise of telemedicine: lessons from a global pandemic. EMJ Innov.

[13] Bokolo Anthony Jnr (2020). Use of telemedicine and virtual care for remote treatment in response to COVID-19 pandemic. J Med Syst.

[14] Anastasiadou D, Folkvord F, Lupiañez-Villanueva F (2018). A systematic review of mHealth interventions for the support of eating disorders. Eur Eat Disord Rev.

[15] Barney A, Buckelew S, Mesheriakova V, Raymond-Flesch M (2020). The COVID-19 pandemic and rapid implementation of adolescent and young adult telemedicine: challenges and opportunities for innovation. J Adolesc Health.

[16] Martin S, Sutcliffe P, Griffiths F, Sturt J, Powell J, Adams A, Dale J (2011). Effectiveness and impact of networked communication interventions in young people with mental health conditions: a systematic review. Patient Educ Couns.

[17] Clus D, Larsen ME, Lemey C, Berrouiguet S (2018). The use of virtual reality in patients with eating disorders: systematic review. J Med Internet Res.

[18] de Carvalho MR, De Santana Dias TR, Duchesne M, Nardi AE, Appolinario JC (2017). Virtual reality as a promising strategy in the assessment and treatment of bulimia nervosa and binge eating disorder: a systematic review. Behav Sci (Basel).

[19] Urrútia G, Bonfill X (2010). Declaración PRISMA: una propuesta para mejorar la publicación de revisiones sistemáticas y metaanálisis. Med Clin (Barc).

[20] Shea BJ, Reeves BC, Wells G, Thuku M, Hamel C, Moran J, Moher D, Tugwell P, Welch V, Kristjansson E, Henry DA (2017). AMSTAR 2: a critical appraisal tool for systematic reviews that include randomised or non-randomised studies of healthcare interventions, or both. BMJ.

[21] Aardoom JJ, Dingemans AE, Spinhoven P, Van Furth EF (2013). Treating eating disorders over the internet: a systematic review and future research directions. Int J Eat Disord.

[22] Yager J (2001). E-mail as a therapeutic adjunct in the outpatient treatment of anorexia nervosa: illustrative case material and discussion of the issues. Int J Eat Disord.

[23] Yager J (2003). E‐mail therapy for anorexia nervosa: prospects and limitations. Euro Eating Disorders Rev.

[24] Cardi V, Lounes N, Kan C, Treasure J (2013). Meal support using mobile technology in anorexia nervosa. Contextual differences between inpatient and outpatient settings. Appetite.

[25] Cardi V, Kan C, Roncero M, Harrison A, Lounes N, Tchanturia K, Meyer C, Treasure J (2012). Mealtime support in anorexia nervosa: a within-subject comparison study of a novel vodcast intervention. Psychother Psychosom.

[26] Treasure J, Macare C, Mentxaka IO, Harrison A (2010). The use of a vodcast to support eating and reduce anxiety in people with eating disorder: a case series. Eur Eat Disord Rev.

[27] Darcy AM, Adler S, Miner A, Lock J (2014). How smartphone applications may be implemented in the treatment of eating disorders: case reports and case series data. Adv Eat Disord.

[28] Juarascio AS, Goldstein SP, Manasse SM, Forman EM, Butryn ML (2015). Perceptions of the feasibility and acceptability of a smartphone application for the treatment of binge eating disorders: qualitative feedback from a user population and clinicians. Int J Med Inform.

[29] Shapiro JR, Bauer S, Andrews E, Pisetsky E, Bulik-Sullivan B, Hamer RM, Bulik CM (2010). Mobile therapy: use of text-messaging in the treatment of bulimia nervosa. Int J Eat Disord.

[30] Hildebrandt T, Michaelides A, Mackinnon D, Greif R, DeBar L, Sysko R (2017). Randomized controlled trial comparing smartphone assisted versus traditional guided self-help for adults with binge eating. Int J Eat Disord.

[31] Bauer S, Okon E, Meermann R, Kordy H (2012). Technology-enhanced maintenance of treatment gains in eating disorders: efficacy of an intervention delivered via text messaging. J Consult Clin Psychol.

[32] Shingleton RM, Pratt EM, Gorman B, Barlow DH, Palfai TP, Thompson-Brenner H (2016). Motivational text message intervention for eating disorders: a single-case alternating treatment design using ecological momentary assessment. Behav Ther.

[33] Hay PJ, Claudino AM (2015). Bulimia nervosa: online interventions. BMJ Clin Evid.

[34] Neumayr C, Voderholzer U, Schlegl S (2020). Psych-APP-therapy: smartphone-based interventions in psychotherapy a systematic review. Verhaltenstherapie.

[35] Losada A, Marmo J (2013). Herramientas de evaluación en trastornos de la conducta alimentaria. Psicología de la Pontificia Universidad Católica Argentina.

[36] Dölemeyer R, Tietjen A, Kersting A, Wagner B (2013). Internet-based interventions for eating disorders in adults: a systematic review. BMC Psychiatry.

[37] Melioli T, Bauer S, Franko DL, Moessner M, Ozer F, Chabrol H, Rodgers RF (2016). Reducing eating disorder symptoms and risk factors using the internet: a meta-analytic review. Int J Eat Disord.

[38] Moghimi E, Davis C, Rotondi M (2021). The efficacy of eHealth interventions for the treatment of adults diagnosed with full or subthreshold binge eating disorder: systematic review and meta-analysis. J Med Internet Res.

[39] Maglia M, Corello G, Caponnetto P (2021). Evaluation of the effects of telepsychotherapy in the treatment and prevention of eating disorders in adolescents. Int J Environ Res Public Health.

[40] Dufour R, Novack K, Picard L, Chadi N, Booij L (2022). The use of technology in the treatment of youth with eating disorders: a scoping review. J Eat Disord.

[41] Zhong J, Zhang Y, Sun Y, Wang Q, Dong G, Li X (2024). The efficacy of internet-based cognitive behavioral therapy for adult binge spectrum eating disorders: a meta-analysis. J Affect Disord.

