# Supplementary material for: Telemedicine in Eating Disorder Treatment: Systematic Review
Source: JMIR Ment Health. 2025 Nov 17;12:e74057. doi: 10.2196/74057 (PMC12670051; doi:10.2196/74057)
Supplement: Multimedia Appendix 1 [file mental_v12i1e74057_app1.docx]

**Search Strategies**

Updated: 2024 December

### Medline (OVID)

1 exp "Feeding and Eating Disorders"/

2 Mental Disorders/dh, dt, nu, px, rh, tu, th [Diet Therapy, Drug Therapy, Nursing, Psychology, Rehabilitation, Therapeutic Use, Therapy]

3 ((eat* or food or feed*) adj2 (disorder* or behabi*)).ti,ab.

4 (anorexi* or bulimi* or (binge adj eat*) or hyperphagia or hypersomnia or (keine adj2 syndrom*) or rumiat* or pica).ti,ab.

5 or/1-4

6 exp Telemedicine/

7 Telenursing/

8 Medical Informatics Applications/

9 computer communication networks/ or internet/

10 telecommunications/ or electronic mail/ or exp telephone/ or videoconferencing/

11 Distance Counseling/

12 Internet-Based Intervention/

13 (e?health or e?consult* or e?therap* or e?intervent* or e?counsel* or m?health).ti,ab

14 ((digital adj2 (health or intervent*)) or (remote adj2 (consult* or therap* or monitor* or assist*))).ti,ab.

15 ((internet or on?line or mobil* or video) adj3 (care or health or consult* or therap* or intervent*)).ti,ab.

16 (telemed* or tele?health or tele?therap* or tele?psychiat* or telepsychol* or tele?care or tele?monitor* or tele?consult*).ti,ab.

17 (video?consult* or video?therap* or video?conferenc*).ti,ab.

18 or/6-17

19 5 and 18

20 meta-analysis.pt.

21 meta-analysis/ or systematic review/ or meta-analysis as topic/ or "meta analysis (topic)"/ or "systematic review (topic)"/ or exp technology assessment, biomedical/

22 ((systematic* adj3 (review* or overview*)) or (methodologic* adj3 (review* or overview*))).ti,ab,kf,kw.

23 ((quantitative adj3 (review* or overview* or synthes*)) or (research adj3 (integrati* or overview*))).ti,ab,kf,kw.

24 ((integrative adj3 (review* or overview*)) or (collaborative adj3 (review* or overview*)) or (pool* adj3 analy*)).ti,ab,kf,kw.

25 (data synthes* or data extraction* or data abstraction*).ti,ab,kf,kw.

26 (handsearch* or hand search*).ti,ab,kf,kw.

27 (mantel haenszel or peto or der simonian or dersimonian or fixed effect* or latin square*).ti,ab,kf,kw.

28 (met analy* or metanaly* or technology assessment* or HTA or HTAs or technology overview* or technology appraisal*).ti,ab,kf,kw.

29 (meta regression* or metaregression*).ti,ab,kf,kw.

30 (meta-analy* or metaanaly* or systematic review* or biomedical technology assessment* or bio-medical technology assessment*).mp,hw.

31 (medline or cochrane or pubmed or medlars or embase or cinahl).ti,ab,hw.

32 (cochrane or (health adj2 technology assessment) or evidence report).jw.

33 (comparative adj3 (efficacy or effectiveness)).ti,ab,kf,kw.

34 (outcomes research or relative effectiveness).ti,ab,kf,kw.

35 ((indirect or indirect treatment or mixed-treatment) adj comparison*).ti,ab,kf,kw.

36 20 or 21 or 22 or 23 or 24 or 25 or 26 or 27 or 28 or 29 or 30 or 31 or 32 or 33 or 34 or 35

37 19 and 36

### EMBASE

#1. 'eating disorder'/exp

#2. 'mental disease'/de

#3. #2 AND ('disease management'/lnk OR 'drug therapy'/lnk OR 'pharmacology'/lnk OR 'rehabilitation'/lnk OR 'therapy'/lnk)

#4. ((eat* OR food OR feed*) NEAR/2 (disorder* OR behabi*)):ti,ab

#5. anorexi*:ti,ab OR bulimi*:ti,ab OR ((binge NEAR/2eat*):ti,ab) OR hyperphagia:ti,ab OR hypersomnia:ti,ab OR ((keine NEAR/2 syndrom*):ti,ab) OR rumiat*:ti,ab OR pica:ti,ab

#6. #1 OR #3 OR #4 OR #5

#7. 'telemedicine'/exp OR 'telehealth'/de OR 'telenursing'/de OR 'computer network'/exp OR 'internet'/de OR 'web-based intervention'/de OR 'e-counseling'/exp OR 'mass communication'/exp

#8. telemed*:ti,ab OR tele$health:ti,ab OR tele$therap*:ti,ab OR tele$psychiat*:ti,ab OR tele$psychol*:ti,ab OR tele$care:ti,ab OR tele$monitor*:ti,ab OR tele$consult*:ti,ab

#9. video$consult*:ti,ab OR video$therap*:ti,ab OR video$conferenc*:ti,ab

#10. ((internet OR on$line OR mobil* OR video) NEAR/3 (care OR health OR consult* OR therap* OR intervent*)):ti,ab

#11. ((digital NEAR/2 (health OR intervent*)):ti,ab) OR ((remote NEAR/2 (consult* OR therap* OR monitor* OR assist*)):ti,ab)

#12. e$health:ti,ab OR e$consult*:ti,ab OR e$therap*:ti,ab OR e$intervent*:ti,ab OR e$counsel*:ti,ab OR m$health:ti,ab

#13. #7 OR #8 OR #9 OR #10 OR #11 OR #12

#14. #6 AND #13

#15. 'meta analysis'/exp OR 'meta analysis topic'/de OR 'meta analysis (topic)'/exp OR 'systematic review'/exp OR 'systematic review (topic)'/exp OR 'biomedical technology assessment'/exp

#16. ((systematic* NEAR/3 (review* OR overview*)):ti,ab,kw) OR ((methodologic* NEAR/3 (review* OR overview*)):ti,ab,kw)

#17. ((quantitative NEAR/3 (review* OR overview* OR synthes*)):ti,ab,kw) OR ((research NEAR/3 (integrati* OR overview*)):ti,ab,kw)

#18. ((integrative NEAR/3 (review* OR overview*)):ti,ab,kw) OR ((collaborative NEAR/3 (review* OR overview*)):ti,ab,kw) OR ((pool*NEAR/3 analy*):ti,ab,kw)

#19. (data NEAR/1 (synthes* OR extraction* OR abstraction*)):ti,ab,kw

#20. handsearch*:ti,ab,kw OR ((hand NEAR/1 search*):ti,ab,kw)

#21. 'matel haenszel':ti,ab,kw OR peto:ti,ab,kw OR 'der simonian':ti,ab,kw OR 'dersimonian':ti,ab,kw OR ((fixed NEAR/1 effect*):ti,ab,kw) OR ((latin NEAR/1 square*):ti,ab,kw)

#22. 'meta analy*':ti,ab,kw OR metanaly*:ti,ab,kw OR 'technology assessment*':ti,ab,kw OR hta:ti,ab,kw OR htas:ti,ab,kw OR ((technology NEAR/1 (overview* OR appraisal*)):ti,ab,kw)

#23. 'meta regression*':ti,ab,kw OR metaregression*:ti,ab,kw

#24. medline:ti,ab,kw OR cochrane:ti,ab,kw OR pubmed:ti,ab,kw OR medlars:ti,ab,kw OR embase:ti,ab,kw OR cinahl:ti,ab,kw

#25. cochrane:jt OR ((health NEAR/2 'technology asseessment'):jt) OR 'evidence report':jt

#26. (comparative NEAR/3 (efficacy OR effectiveness)):ti,ab,kw

#27. 'outcomes reseach':ti,ab,kw OR 'relative effectiveness':ti,ab,kw

#28. ((indirect OR 'indirect treatment' OR 'mixed-treatment') NEAR/1 comparison*):ti,ab,kw

#29. #15 OR #16 OR #17 OR #18 OR #19 OR #20 OR #21 OR #22 OR #23 OR #24 OR #25 OR #26 OR #27 OR #28

#30. #14 AND #29

#31. #30 AND [embase]/lim NOT ([embase]/lim AND [medline]/lim)

#32. #30 AND [embase]/lim NOT ([embase]/lim AND [medline]/lim) NOT 'conference abstract'/it

###

### Web of Science

14 **(#3 AND #9) AND TS=((systematic NEAR1 review) Or meta$analysis)** and **MEDLINE®** (Exclude – Database)

13 **(#3 AND #9)** and **MEDLINE®** (Exclude – Database)

12 **(#3 AND #9)**

11 **(#3 AND #9) AND TS=((systematic NEAR1 review) Or meta$analysis)**

10 **#3 AND #9**

9 **#4 OR #5 OR #6 OR #7 OR #8**

8 **TS=((e$health or e$consult* or e$therap* or e$intervent* or e$counsel* or m$health))**

7 **TS=(((digital NEAR/2 (health or intervent*)) or (remote NEAR/2 (consult* or therap* or monitor* or assist*))))**

6 **TS=((internet or on$line or mobil* or video) NEAR/3 (care or health or consult* or therap* or intervent*))**

5 **TS=(video$consult* or video$therap* or video$conferenc*)**

4 **TS=(telemed* or tele$health or tele$therap* or tele$psychiat* or tele$psychol* or tele$care or tele$monitor* or teleconsult*)**

**3 #1 OR #2**

**2 TS=(anorexi* or bulimi* or (binge NEAR/1 eat*) or hyperphagia or hypersomnia or (keine NEAR/2 syndrom*) or rumiat* or pica)**

**1 TS=(((eat* or food or feed*) NEAR/2 (disorder* or behabi*)))**

### Cochrane library

#32 MeSH descriptor: [Feeding and Eating Disorders] explode all trees

#33 MeSH descriptor: [Mental Disorders] explode all trees and with qualifier(s): [diet therapy - DH, drug therapy - DT, nursing - NU, psychology - PX, rehabilitation - RH, therapy - TH]

#34 ((eat* or food or feed*) NEAR/2 (disorder* or behabi*)):ti,ab

#35 (anorexi* or bulimi* or (binge NEAR/1 eat*) or hyperphagia or hypersomnia or (keine NEAR/2 syndrom*) or rumiat* or pica):ti,ab

#36 #32 Or #33 OR #34 Or #35

#37 MeSH descriptor: [Telemedicine] explode all trees

#38 MeSH descriptor: [Telenursing] explode all trees

#39 MeSH descriptor: [Medical Informatics Applications] explode all trees

#40 MeSH descriptor: [Computer Communication Networks] explode all trees

#41 MeSH descriptor: [Internet] explode all trees

#42 MeSH descriptor: [Telecommunications] explode all trees

#43 MeSH descriptor: [Electronic Mail] explode all trees

#44 MeSH descriptor: [Telephone] explode all trees

#45 MeSH descriptor: [Videoconferencing] explode all trees

#46 MeSH descriptor: [Distance Counseling] explode all trees

#47 MeSH descriptor: [Internet-Based Intervention] explode all trees

#48 (telemed* or tele?health or tele?therap* or tele?psychiat* or telepsychol* or tele?care or tele?monitor* or tele?consult*):ti,ab

#49 (video?consult* or video?therap* or video?conferenc*):ti,ab

#50 ((internet or on?line or mobil* or video) NEAR/3 (care or health or consult* or therap* or intervent*)):ti,ab

#51 ((digital NEAR/2 (health or intervent*)) or (remote NEAR/2 (consult* or therap* or monitor* or assist*))):ti,ab

#52 (e?health or e?consult* or e?therap* or e?intervent* or e?counsel* or m?health):ti,ab

#53 #37 Or #38 OR #39 Or #40 OR #41 OR #42 OR #43 OR #44 OR #45 OR #46 Or #47 OR #48 OR #49 OR #50 OR #51 Or #52

#54 #36 AND #53 in Cochrane Reviews, Cochrane Protocols

### CINAHL

S1 (MH "EatingDisorders+")

S2 (MH "Mental Disorders/DH/DT/NU/RH/TH/PF")

S3 TI ( ((eat* or food or feed*) N2 (disorder* or behabi*)) ) OR AB ( ((eat* or food or feed*) N2 (disorder* or behabi*)) )

S4 TI ( (anorexi* or bulimi* or (binge N1 eat*) or hyperphagia or hypersomnia or (keine N2 syndrom*) or rumiat* or pica) ) OR AB ( (anorexi* or bulimi* or (binge N1 eat*) or hyperphagia or hypersomnia or (keine N2 syndrom*) or rumiat* or pica) )

S5 (S1 OR S2 OR S3 OR S4)

S6 (MH "Telehealth") OR (MH "Telemedicine+") OR (MH "Telenursing") OR (MH "Telecommunications")

S7 (MH "Medical Informatics")

S8 (MH "Computer Communication Networks") OR (MH "Internet+")

S9 (MH "Videoconferencing") OR (MH "Telephone+") OR (MH "Telehealth")

S10 (MH "RemoteConsultation")

S11 TI ( (telemed* or tele#health or tele#therap* or tele#psychiat* or tele#psychol* or tele#care or tele#monitor* or tele#consult*) ) OR AB ( (telemed* or tele#health or tele#therap* or tele#psychiat* or tele#psychol* or tele#care or tele#monitor* or tele#consult*) )

S12 TI ( (video#consult* or video#therap* or video#conferenc*) ) OR AB ( (video#consult* or video#therap* or video#conferenc*) )

S13 TI ( ((internet or on#line or mobil* or video) N3 (care or health or consult* or therap* or intervent*)) ) OR AB ( ((internet or on#line or mobil* or video) N3 (care or health or consult* or therap* or intervent*)) )

S14 TI ( ((digital N2 (health or intervent*)) or (remote N2 (consult* or therap* or monitor* or assist*))) ) OR AB ( ((digital N2 (health or intervent*)) or (remote N2 (consult* or therap* or monitor* or assist*))) )

S15 TI ( (e#health or e#consult* or e#therap* or e#intervent* or e#counsel* or m#health) ) OR AB ( (e#health or e#consult* or e#therap* or e#intervent* or e#counsel* or m#health) )

S16 (S6 OR S7 OR S8 OR S9 OR S10 OR S11 OR S12 OR S13 OR S14 OR S15)

S17 (S5 AND S16)

S18 (((ZT "systematic review")) or ((ZT "review"))) or ((ZT "meta analysis"))

S19 (S17 AND S18)

### INAHTA

("Telemedicine"[mh]) AND ("Feeding and Eating Disorders"[mh]) = 0

telemedicine and anorexia = 0

telemedicine and bulimia =0

### PSYCINFO

S1 DE "EatingDisorders"

S2 TI ( ((eat* or food or feed*) N2 (disorder* or behabi*)) ) OR AB ( ((eat* or food or feed*) N2 (disorder* or behabi*)) )

S3 TI ( (anorexi* or bulimi* or (binge N1 eat*) or hyperphagia or hypersomnia or (keine N2 syndrom*) or rumiat* or pica) ) OR AB ( (anorexi* or bulimi* or (binge N1 eat*) or hyperphagia or hypersomnia or (keine N2 syndrom*) or rumiat* or pica) )

S4 (S1 OR S2 OR S3)

S5 (((((((DE "Electronic Health Services") OR (DE "Computer Assisted Therapy")) OR (DE "Telemedicine" OR DE "Online Therapy" OR DE "Teleconferencing" OR DE "Teleconsultation" OR DE "Telepsychiatry" OR DE "Telepsychology" OR DE "Telerehabilitation")) OR (DE "Electronic Communication" OR DE "Computer Mediated Communication" OR DE "Social Media" OR DE "Text Messaging")) OR (DE "Internet")) OR (DE "Digital Interventions")) OR (DE "Online Therapy")) OR (DE "Video-Based Interventions")

S6 TI ( (telemed* or tele#health or tele#therap* or tele#psychiat* or tele#psychol* or tele#care or tele#monitor* or tele#consult*) ) OR AB ( (telemed* or tele#health or tele#therap* or tele#psychiat* or tele#psychol* or tele#care or tele#monitor* or tele#consult*) )

S7 TI ( (video#consult* or video#therap* or video#conferenc*) ) OR AB ( (video#consult* or video#therap* or video#conferenc*) )

S8 TI ( ((internet or on#line or mobil* or video) N3 (care or health or consult* or therap* or intervent*)) ) OR AB ( ((internet or on#line or mobil* or video) N3 (care or health or consult* or therap* or intervent*)) )

S9 TI ( ((digital N2 (health or intervent*)) or (remote N2 (consult* or therap* or monitor* or assist*))) ) OR AB ( ((digital N2 (health or intervent*)) or (remote N2 (consult* or therap* or monitor* or assist*))) )

S10 TI ( (e#health or e#consult* or e#therap* or e#intervent* or e#counsel* or m#health) ) OR AB ( (e#health or e#consult* or e#therap* or e#intervent* or e#counsel* or m#health) )

S11 (S5 OR S6 OR S7 OR S8 OR S9 OR S10)

S12 (S4 AND S11)

S13 ((ZC "literature review") or (ZC "meta analysis")) or ((ZC "systematic review"))

S14 (S12 AND S13)
