# Supplementary material for: Telemedicine in Eating Disorder Treatment: Systematic Review
Source: JMIR Ment Health. 2025 Nov 17;12:e74057. doi: 10.2196/74057 (PMC12670051; doi:10.2196/74057)
Supplement: Multimedia Appendix 3 [file mental_v12i1e74057_app3.docx]

**Qualitative findings**

Table A3. Qualitative findings of telemedicine intervention by patients ‘condition.

| **Condition** | **Author, year** | **Intervention** | **Qualitative information** |
| --- | --- | --- | --- |
|  |  |  |  |
| Anorexia nervosa | Aardoom, 2013 [23] | Email | Patients positively experienced the use of email and generally found it helpful.  E-mail contact was encouraging and motivating. A good way to stay in touch with the therapist is to become aware of eating behaviours and problems. |
|  | Anastasiadou, 2018 [14] | Vodcast | The vodcast is not very useful; too focused on eating disorder issues (10 users). |
|  |  | Vodcasts: energy controller, mindful eating, motivation, and reflection | Useful and accessible.  Increase average intake (grams).  Reduction of anxiety (3 out of a total of four patients).  Increase in weight three months later.  Helpful and positive feedback on content.  Support and motivation. |
|  | Martin, 2011 [16] | Email | Email increases the frequency and amount of direct contact between patients and physicians. Patients spend a lot of time composing emails for physicians.  Some patients described having "virtually constant access to their physicians".  Email allows patients to express themselves more easily.  Electronic submission of calorie counts, or symptom records frees up time in face-to-face sessions.  Concern about unwanted disclosures to third parties stemming from a lack of computer privacy. |
| Bulimia nervosa | Anastasiadou, 2018 [14] | SMS^g^ text messaging: BN^d^ symptom self-monitoring Program with CBT^e^ Components | Attrition rate: 48.4% of the total sample and 60% of those who started.  High retention rate: 87%.  Acceptability: above average. |
| Bing Eating Disorder | Anastasiadou, 2018 [14] | Smartphone app. BED^c^ Self-Help Program | Feasible and acceptable application.  Concern for customization and adaptability.  Concerns about privacy and sharing.  Potential efficacy to help reduce binge eating episodes. |
| Heterogeneous population | Aardoom, 2013 [23] | Population: AN^a^ and ED^f^  Email | Patients positively experienced the use of email and generally found it useful. For example, participants indicated that they found email contact encouraging and motivating, that it was a good way to stay in touch with their therapist, and that it made them aware of their eating behaviours and problems. |
|  | Anastasiadou, 2018 [14] | Population: AN^a^ and BN  Smartphone App^b^: “*Food For Thought*” | Accessibility and feasibility.  Increase frequency of food records using the app compared to paper and pen records.  Wide range of applications.  Acceptable, easy to use.  Covers a broad spectrum of patients. |
|  |  | Population: BN^d^ and BED^c^  Smartphone App^b^: “*Noom Monitor*” | Adherence and alliance were the same in both conditions. |
|  |  | Population: AN^a^ and BN^d^  SMS^g^ text Messages | Feasibility: 91.5% of daily entries and 87.4% of daily food records.  Acceptability: mean (SD^h^) = 7.05 (2.36) out of 10 points.  High retention rate: 92.2%.  Participants liked the intervention and the usefulness of the reminders.  Some patients are not satisfied with the personalized nature of the messages. |
|  | Martin, 2011 [16] | Population: AN^a^ and ED^f^ undefined  Email | No patients reported significant adverse effects from email.  E-mail increases the frequency and time of contact between patients, clinicians and therapeutic processes. There is positive emotional value because of communication, when needed.  Near-daily email reports require patients to be constantly aware of their behaviours and of being in therapy.  Providing details via email frees up time in face-to-face sessions. |

^a^AN, anorexia nervosa;

^b^App, smartphone application

^c^BED, binge eating disorder

^d^BN, bulimia nervosa

^e^CBT, cognitive behavioural therapy

^f^ED, eating disorder

^g^SMS, short message service;

^h^SD, standard deviation.
